# Assessment of Immunogenicity and Efficacy of a Zika Vaccine Using Modified Vaccinia Ankara Virus as Carriers

**DOI:** 10.3390/pathogens8040216

**Published:** 2019-11-02

**Authors:** César López-Camacho, Young Chan Kim, Peter Abbink, Rafael A. Larocca, Juha T. Huiskonen, Dan H. Barouch, Arturo Reyes-Sandoval

**Affiliations:** 1 The Jenner Institute, Nuffield Department of Medicine, University of Oxford, The Henry Wellcome Building for Molecular Physiology, Roosevelt Drive, Oxford OX3 7BN, UK; 2 Division of Structural Biology, University of Oxford, Wellcome Centre for Human Genetics, Roosevelt Drive, Headington, Oxford OX3 7BN, UK; 3 Center for Virology and Vaccine Research, Beth Israel Deaconess Medical Center, Harvard Medical School, Boston, MA 02215, USA

**Keywords:** Zika virus, MVA, envelope proteins, vaccines, immunogenicity, efficacy, mice

## Abstract

Zika virus (ZIKV) is an emerging mosquito-borne flavivirus that has spread to more than 70 countries worldwide since 2015. Despite active research, there are currently no licensed vaccines or therapeutics. We have previously reported the development of various adenoviral vectored vaccine candidates (ChAdOx1 ZIKV) with the ability to stimulate effective immunity in mice and provide protection upon a ZIKV challenge model, using a non-adjuvanted single vaccination approach. In this study, we constructed various modified vaccinia Ankara (MVA) viruses to express the ZIKV Envelope (E) with modifications on the precursor membrane (prM) or on the C-terminus envelope transmembrane domain (TM), similar to our ChAdOx1 vaccine candidates. MVA-ZIKV vaccine candidates were evaluated as a non-adjuvanted single vaccination regimen against a ZIKV Brazilian isolate, using viraemia as the correlate of protection. Here, we report the induction of a modest level of anti-ZIKV E antibodies by all MVA vectored vaccines and sub-optimal efficacy in a ZIKV challenge model. Our results indicate the requirement of additional strategies when using MVA-ZIKV vaccines to afford sterile protection upon a non-adjuvanted and single vaccination regime.

## 1. Introduction

Zika virus (ZIKV) is a mosquito-borne flavivirus virus that belongs to the family *Flaviviridae* [1]. After its first discovery in 1947 from a sentinel rhesus monkey in Uganda, ZIKV caused sporadic outbreaks in Africa and South Asia until the occurrence of major outbreaks in Micronesia in 2007 and French Polynesia in 2013 [2,3]. ZIKV has spread rapidly throughout the Americas since its first report in Brazil in 2015 [4], affecting more than 70 countries worldwide [5]. ZIKV is classified into two lineages: African (AF) and Asian (AS) [6]. The Asian lineage is causing the current outbreaks occurring worldwide. The main vector for urban transmission of ZIKV is the *Aedes* mosquito, although sexual contact and vertical transmission are also responsible for the virus dissemination [7]. Infection by ZIKV is associated with neurological complications, such as microcephaly in foetuses and Guillain–Barré syndrome (GBS) in adults, now considered congenital zika syndrome (CZS) [8,9,10]. There has been considerable progress in the research of vaccines or therapeutics against ZIKV, however, no licensed vaccines are yet available against ZIKV. There are many ZIKV vaccine candidates, such as inactivated virus, based on DNA, mRNA, and recombinant viral vectors encoding the precursor membrane (prM) and the envelope (E) ZIKV proteins, which are currently in phase I or II clinical trials [11]. The modified vaccinia virus Ankara (MVA) has been extensively studied as a vectored-vaccine against various infectious diseases, reaching clinical trials, where it has been regarded as a safe, cost effective, and efficacious vaccine vector [12,13,14,15,16]. We have previously reported the development of four ChAdOx1 ZIKV vaccine candidates and their protective efficacy in a homologous ZIKV challenge model [17]. All four ChAdOx1 ZIKV vaccine candidates (prME, prME ΔTM, Env, and Env ΔTM) were shown to stimulate the production of anti-E ZIKV antibodies and demonstrated protective efficacy in a homologous ZIKV-lineage challenge model. The vaccine candidate that contains prME and has a deletion (Δ) in the transmembrane domain (prME ΔTM) induced the highest titres of anti-envelope ZIKV antibodies that provided 100% efficacy against ZIKV infection, with only a single and non-adjuvanted vaccination. Here, we describe the development of MVA-ZIKV vaccine candidates based on the same Asian lineage sequence as our previous ChAdOx1-ZIKV vaccine candidates. All the MVA-ZIKV vaccines induced modest levels of anti-ZIKV envelope antibodies measured at 4 weeks and 12 weeks post-immunisation. In a ZIKV mice challenge model, two MVA-ZIKV vaccine candidates (Env ΔTM and prME) provided the best, yet partial protection against ZIKV, as shown by the reduction in levels of viraemia in all BALB/c mice, while the rest of the MVA candidates offered a lower degree of reduction in viral load in mice. This study reports that MVA ZIKV vaccine candidates may be a limited candidate for further clinical assessment, if used as a single-vaccination approach.

## 2. Results

### 2.1. Modified Vaccinia Ankara (MVA) Expressing ZIKV Antigens

**Figure 1:**
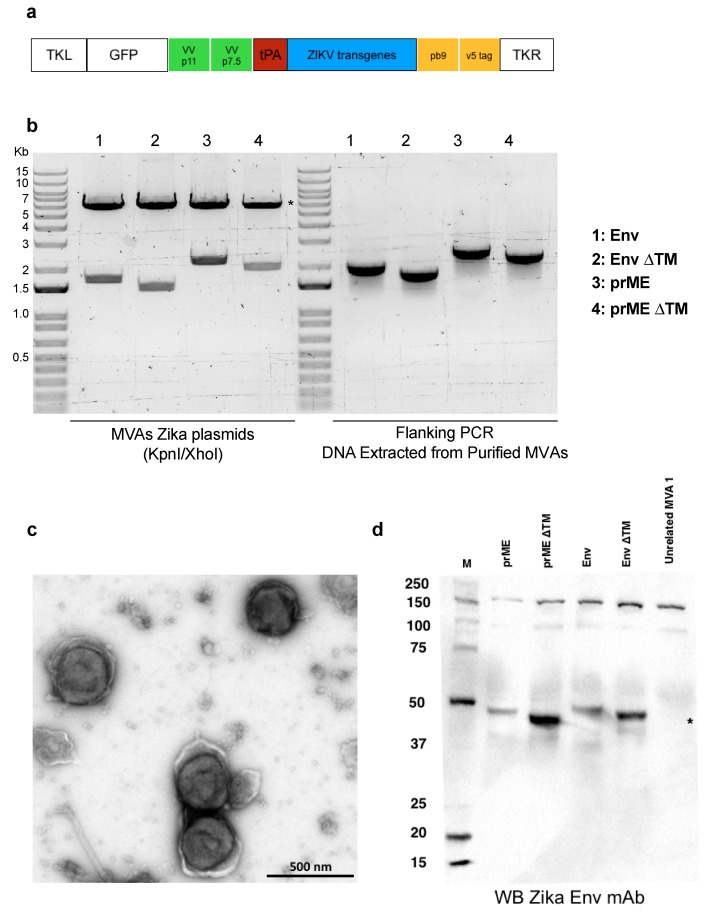
MVA-ZIKV vaccine designs. (**a**) Schematic representation of the genetic cassette used to produce the recombinant MVA vectors, containing the ZIKV structural genes shown in blue box. ((**b**), left panel) Enzyme restriction analysis of the MVA-ZIKV plasmids used to construct the recombinant viral vectors. Restriction released the tPA leading sequence (red box in Figure 1a) plus each of the ZIKV transgenes. Asterisks denote the MVA backbone plasmid. ((**b**), right panel) Flanking PCR analysis from DNA-purified virus to confirm the presence of the antigen expression cassette. (**c**) Transmission electron microscopy of purified MVA prME ΔTM preparation. Sucrose-purified virions were negative stained and processed for electron microscopy. Typical brick-shaped particles were detected [18]. Bar, 500 nm. (**d**) Expression of ZIKV immunogens by western blot of BHK21 cell extracts transduced with MVA-ZIKV vaccine candidates, using an anti-ZIKV E antibody as the primary antibody. A change in size can be appreciated in the monomeric form of ZIKV E (around 45 kDa for full length E, in comparison with the TM deleted version of E), asterisk.

To generate MVA-based ZIKV vaccine candidates, we sub-cloned each of the ZIKV transgenes (prME, prME ΔTM, Env, Env ΔTM) with parental MVA plasmid (Figure 1a). After transfection of AatII-restriction enzyme linearised MVA-ZIKV plasmids, MVA particles were extracted and purified. DNA extraction from purified MVA vaccines was carried out to verify the correct transgene DNA length. The correct generation of all MVA-ZIKV vaccine candidates was verified by PCR, using flanking regions (primer p7.5 and primer TKR), confirming the integrity of the transgenes within the MVA-ZIKV genomes (Figure 1b). To ensure the viral preparation was satisfactory, we tested the MVA-ZIKV vaccine candidate expressing the prME ΔTM, under negative stain and transmission electron microscopy (TEM), confirming the brick-shaped morphology and size (~305 nm by 260 nm) expected of the MVA vector, as described elsewhere [18] (Figure 1c). The expression of the ZIKV immunogens was confirmed by western blot in cells extracts from MVA-ZIKV-transduced BHK21 cells; using an anti-ZIKV E primary antibody (Figure 1d). Taken together, we showed that MVA viral vectors are correctly packaged, carrying the ZIKV transgenes in their genome and more importantly, that they are capable of inducing the expression of the ZIKV E in transduced cells.

### 2.2. Assessment of Immunogenicity

**Figure 2:**
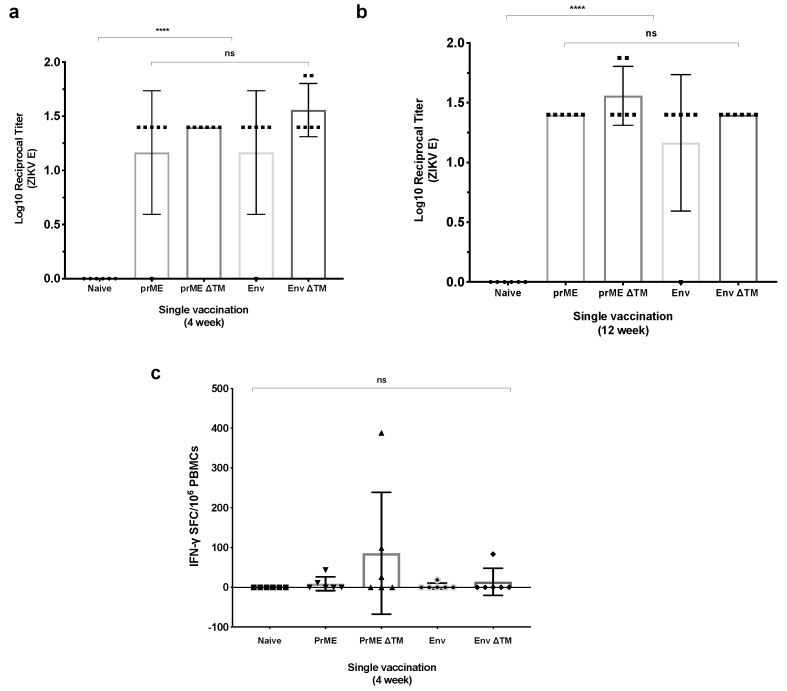
Immune responses elicited by MVA-ZIKV vaccines. BALB/c mice (*n* = 6) were intramuscularly immunised with a single dose of MVA encoding ZIKV antigens at 1 × 10^6^ plaque forming units (PFU)/mouse. Serum samples were collected at 4 weeks and 12 weeks post immunisation. (**a**) Antibody responses elicited by MVA-ZIKV vaccine candidates at 4 weeks and (**b**) 12 weeks post immunisation were quantified by ELISA in plates coated with a ZIKV E protein [19]. (**c**) Cellular immune responses to MVA-ZIKV vaccine candidates. PBMCs IFNγ–producing cells after 4 weeks post immunisation were measured by (IFNγ) ex vivo ELISPOT, and 20-mer peptides spanning the ZIKV prME proteins at 10 μg/mL were used for stimulation. *p*-values were determined by one-way ANOVA and Tukey’s multiple comparisons test.

We assessed the immunogenicity of our MVA-ZIKV vaccine candidates by monitoring the anti-ZIKV E antibody titres, 4 weeks and 12 weeks after a non-adjuvanted and single intramuscular vaccine immunisation in BALB/c mice (n = 6), by enzyme-linked immunosorbent assay (ELISA). At four weeks post-immunisation, all MVA-ZIKV vaccine candidates induced the production of anti-ZIKV E antibodies, with an antibody increase trend in the groups in which the vaccines were lacking the E transmembrane domain (TM) (Figure 2a). At this timepoint, MVA Env ΔTM induced the highest level of antibodies (mean endpoint titre = 1.56). At 12 weeks post-prime immunisation, the amount of anti-ZIKV E titres elicited by the MVA vaccine candidates showed a non-statistically significant increase between vaccinated groups, with the prME ΔTM group having the highest mean log reciprocal titre of 1.55 (Figure 2b). Anti-ZIKV E antibodies, in sera from the naïve group, were negative at any measured timepoint. Furthermore, we assessed cellular responses from isolated peripheral blood mononuclear cells (PBMCs), elicited by MVA-ZIKV vaccine candidates after four weeks post-immunisation. Interferon-γ (IFNγ) enzyme-linked immunospot (ELISPOT) assay showed that three out of six BALB/c mice receiving MVA prME ΔTM induced modest IFNγ-producing T cell responses (mean = 85.5 spot forming units (SFU)/10^6^ PBMCs), while the rest of vaccine candidates induced smaller amount of IFNγ-producing T cell responses (mean < 20 SFU/10^6^ PBMCs). Taken together, MVA-ZIKV vaccines are immunogenic in BALB/c mice and humoral responses remained similar between 4 to 12 weeks after a single vaccination dose, whereas low levels of cellular responses were detected at 4 weeks post-immunisation.

### 2.3. ZIKV Challenge in BALB/c Mice

**Figure 3:**
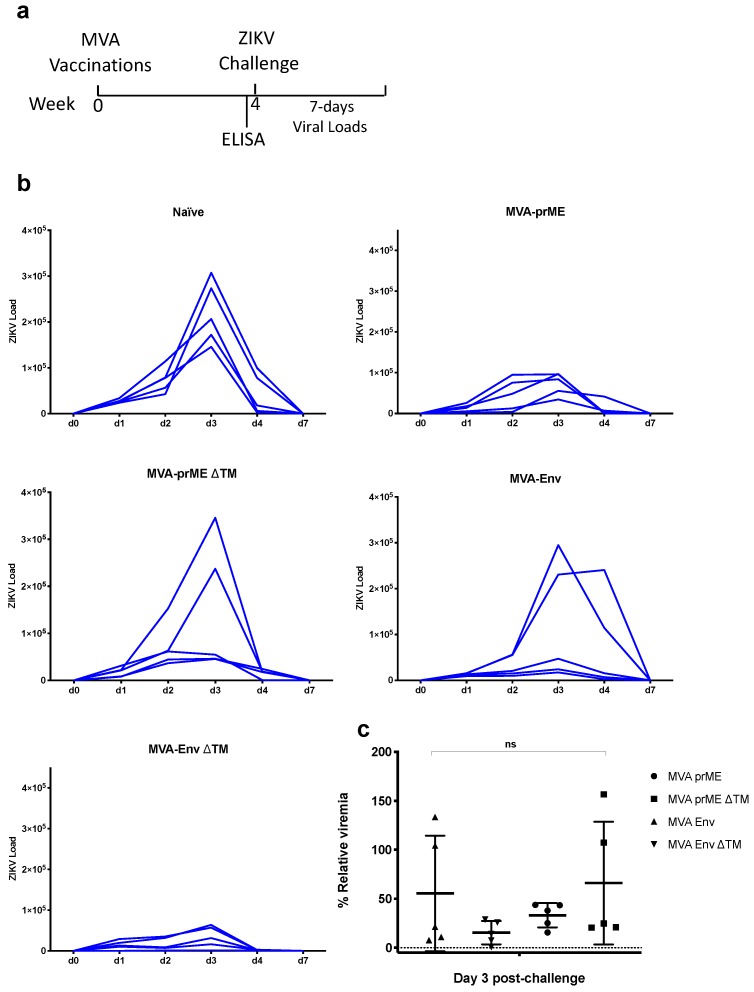
Assessment of protective efficacy induced by MVA-ZIKV vaccines. (**a**) Naïve and vaccinated BABL/c mice (*n* = 5) were intravenously challenged with 100 PFU of ZIKV-BR strain, at four weeks post immunisation. After ZIKV challenge, viral loads were monitored for seven days. (**b**) Viral load kinetics in ZIKV-challenged groups were monitored to assess the protective efficacy. Graphs show days post-challenge on the *x*-axis versus viral loads on the *y*-axis. Continuous blue lines represent one mouse each, for each of the groups. (**c**) Relative viraemia at day 3 post-challenge, for each individual mouse and for each group was calculated based in the mean viremia from the naïve mice group. *p*-values were determined by one-way ANOVA and Tukey’s multiple comparisons test.

To assess the efficacy of the our MVA-ZIKV vaccine candidates, BALB/c mice at four-weeks post-immunisation (*n* = 5) were intravenously challenged with ZIKV (strain Brazil ZKV2015; ZIKV-BR), as previously described [17]. Vaccine efficacy was measured as partial or complete reduction of viremia, by retro-transcriptase (RT)-quantitative polymerase chain reaction (qPCR); at 0, 1, 2, 3, 4, and 7-days post ZIKV challenge (Figure 3). The naïve control group challenged with ZIKV displayed the expected viraemia kinetics, comprising a viral load peak at day 3 and clearance of viraemia by day 7 (Figure 3a, naïve panel). Mice vaccinated with a single dose of MVA-ZIKV vaccines conferred partial protection against ZIKV challenge. For the MVA prME vaccinated group, all mice reduced viral loads in the blood: reduced viral load peak was detected in three out of five mice, and the other two mice presented a viral load peak at day 2, instead of day 3. After the viral peak, all mice dropped viral loads by day 4 and cleared the virus by day 7. Mice receiving the MVA prME ΔTM vaccine reflected lower viraemia in three out of five mice, whereas two mice showed viraemia similar to that of the control naïve group. A similar trend was observed in the MVA vaccine expressing only E (MVA-Env). Interestingly, the MVA vaccine expressing E without the TM domain (MVA Env ΔTM), improved protective efficacy, in comparison to the rest of the vaccinated groups. Besides that, the protection efficacy of MVA Env ΔTM was better than the rest of the MVA-ZIKV vaccines, by substantially reducing the ZIKV loads in blood, such protection did not reach 100% efficacy and we did not detect statistical significance between the MVA-ZIKV vaccinated groups when analysing three-day post challenge viral loads. (Figure 3c). Taken together, we conclude that a single dose and non-adjuvanted vaccination with the MVA-ZIKV vaccines elicited humoral immunogenicity, but these vaccines could not afford sterile protection in BALB/c mice challenged with ZIKV.

## 3. Discussion

A ZIKV vaccine target product profile [20] indicates that a preferred dose regimen in a Zika vaccine development is a single immunisation, although a minimal requirement of more than one dose is still a possibility. However, the challenge remains of ensuring protective efficacy in prime-boost regimens using a short vaccination interval, with the added burden of duplicating the administration cost, its delivery, dose, storage, etc.

We have previously demonstrated that a non-replicative adenoviral-vectored vaccine expressing ZIKV antigens induces high levels of immunity and protective efficacy in a ZIKV mice challenge model against a ZIKV-BR strain, all upon an adjuvanted, single adenoviral-based vaccine [17]. Here, we explored the suitability of different viral-vectored platforms other than adenoviral-vectored vaccines; the modified vaccinia Ankara (MVA) virus was engineered to express the same antigens that were previously expressed in adenoviral-vectored vaccines. The rationale behind constructing MVA vectored-ZIKV vaccines relies in the proven safety in clinical trials and its high immunogenicity demonstrated in various vaccine developments. Furthermore, Pérez et al. constructed an MVA-ZIKV vaccine expressing the *prME* gene, which is capable of expressing Virus-Like Particles (VLPs) and inducing partial protection in mice after a ZIKV challenge, upon a homologous prime-boost approach [21]. Of particular interest, an MVA vaccine expressing the non-structural protein 1 (NS1) of ZIKV has been demonstrated to be 100% effective in both a single- or two-dose vaccination [22]. Here, we developed four MVA-ZIKV vaccines expressing the envelope gene, with or without its transmembrane domain, both in the presence and absence of the prM antigens. In our hands, the use of MVA was not as efficient as the ChAdOx1 counter-parts [17]. MVA-ZIKV vaccine candidates induced modest levels of anti-ZIKV E antibodies after a single vaccination approach and they failed to confer sterile protection. This observation agrees with Perez et al., in which a single immunisation of MVA-ZIKV induced a 2.5 log reduction in viraemia, whereas a two-dose immunisation further decreased the viral load but no sterile protection was achieved [21]. These data highlight the following considerations. The sub-optimal protection observed in our study might improve if a booster vaccine is provided. If the elicitation of anti-ZIKV antibodies is key to confer 100% sterile protection, then there is a need to improve the vaccine dose and its delivery, taking into consideration the intervals of vaccination to mount and maintain a long-lasting response against ZIKV. Because our aim was to develop a protective vaccine, given in a single administration, we conclude that the MVA-ZIKV vaccines did not ensure full protective efficacy. However, it would be of interest to assess a two-dose regimen, particularly for the MVA Env ΔTM, the vaccine that performed the best at controlling viraemia. Furthermore, it would be interesting to test a combinatorial prime-boost strategy between the MVA-ZIKV vaccines (i.e., a prime with MVA-prME followed by a booster with MVA Env ΔTM), with the objective to assess the best combination that could improve the results observed by us and others. In this study, we used a BALB/c mouse strain that had no lethal outcome upon ZIKV infection. Hence, we measured vaccine efficacy by means of partial or complete reduction of viraemia by RT-qPCR. However, further studies, such as the use of a highly susceptible ZIKV challenge model in A129, mice would be helpful to explore the ZIKV presence in key organs, such as the brain, spleen, and reproductive tract. Given the level of efficacy of MVA-ZIKV vaccines in preventing viraemia, it is expected that ZIKV viral loads may be found in those organs. Lastly, MVA vaccines have been shown to be remarkably efficient when given as a heterologous boost [23]. Therefore, a vaccination given in a prime-boost regimen (ChAdOx1/MVA) could be considered to increase the levels of neutralising antibodies against ZIKV, although a ChAdOx1 prME ΔTM vaccine has been shown to elicit functional and long-lasting antibodies up to 9 months and 13 months after a single immunisation in inbred and outbred mice, respectively [17]. Various ZIKV vaccine platforms based on the ZIKV envelope have been reported, which consist of DNA, mRNA, VLPs or adenoviral vectors, MVAs, and using different routes of administrations. Therefore, levels of immunogenicity, ZIKV neutralisation, or in vivo efficacy outcomes are still a good standard to assess such vaccine developments. Our results provide valuable information towards the development of an efficacious vaccine development that could prevent future ZIKV outbreaks.

## 4. Materials and Methods

Female inbred BALB/c mice were used for the assessment of immunogenicity and ZIKV challenge. Mice were purchased from either Envigo (Bicester, UK) or from Charles River (Wilmington, MA, USA).

All animals and procedures were used in accordance with the terms of the UK Home Office Animals Act Project License. Immunisation and immunogenicity procedures were approved by the University of Oxford Animal Care and Ethical Review Committee (P9804B4F1). Animal experiments performed at Harvard (immunisation and ZIKV challenge) were approved by the BIDMC Institutional Animal Care and Use Committee (IACUC).

The transgene was designed based on the Asian Lineage and produced by GeneArt (prME). The GeneArt prME plasmid was used as template to further generate the four ZIKV antigens (prME, prME ΔTM, Env, and Env ∆TM) as previously described ([17]). ZIKV antigens were used to generate MVA viruses as previously described [24,25]. Briefly, the resulting antigen sequences were ligated into a shuttle vector, pMVA-GFP, which placed the open reading frame (ORF) under the control of the vaccinia p7.5 early/late promoter and also included GFP as a marker gene under the control of the vaccinia p11 late promoter. The shuttle vector was transfected into chicken embryo fibroblasts (CEF) infected with MVA for homologous recombination and insertion of the ZIKV ORF and GFP marker gene at a TK locus of MVA. The infected cells were harvested and screened by PCR to confirm identity. Plaque picking was performed until the culture was free of parental virus, as determined by PCR. The recombinant viruses were plaque purified and then expanded in Doug-Foster-1 (DF-1) cells [24].

The preparation of MVA vaccine stocks was performed by the infection of permissive CEF cells at low multiplicity of infection (M.O.I.). The supernatants were collected and the remaining cells lysed by successive rounds of freezing and thawing. The released virus was further concentrated by centrifugation through 36% (*w*/*v*) sucrose cushions and titres determined by routine plaque assay in DF-1 cell monolayers [24].

Formvar/carbon 200 Mesh Cu grids were glow-discharged in air and loaded with 3.0 μL of MVA viral sample encoding prME ΔTM. Excess liquid was removed, and the grids were washed three times with MilliQ water. Finally, grids were treated with 2% uranyl acetate for 30 s, excess uranyl acetate was carefully removed using filter paper. The grids were air dried and analysed with a T12 transmission electron microscope (FEI, Eindhoven, The Netherlands).

To test the expression of ZIKV antigens, BHK-21 cells were transfected with rMVA. At 24 h after transfection, BHK-21 cell extracts were tested by Western blot as previously described [19]. Briefly, samples added to Laemmli sample buffer containing 20 mM β-mercaptoethanol, boiled for 5 min, and loaded on a Mini-PROTEAN TGX protein gel with Bio-Rad, Precision Plus Protein^TM^ WesternC^TM^ standards. Proteins were transferred onto nitrocellulose membranes (Bio-Rad Trans-Blot^®^ Turbo^TM^). The membranes were blocked with 5% skimmed milk in PBS/ 0.1% Tween (PBST) for 1 h and then incubated with 1:1000 anti-Zika Env monoclonal antibody (mouse mAb to Zika Env protein, AZ1176, Aalto BioReagents, Dublin, Republic of Ireland). After washing with PBST, membranes were incubated for 1 h with an appropriate secondary antibody using an HRP-conjugated goat anti-mouse IgG (Bio-Rad Cat. 170-6516). The membranes were washed and incubated with chemiluminescent substrate (Clarity^TM^ Western ECL Blotting Substrates, Bio-Rad, Watford, UK) prior to detecting the signal by the chemiluminescent Western blot imaging system (Image Lab, Bio-Rad).

Groups of five or six mice were vaccinated with a single dose of new recombinant modified vaccinia ankara (MVA) encoding the ZIKV antigens at a dose of 1 × 10^6^ PFU. All viral vector vaccines were administered intramuscularly (IM) and diluted in endotoxin-free PBS.

Enzyme-linked immunosorbent assay (ELISA)-recombinant ZIKV Envelope protein was used to measure anti-ZIKV envelope antibody concentrations in vaccinated mice by ELISA as previously described [19]. Briefly, Nunc Maxisorp Immuno ELISA plates were coated with Zika virus envelope antigen (Env-CD4) diluted in PBS to a final concentration of 2 μg/mL and left at room temperature (RT )overnight. Plates were washed six times with PBS/0.05% Tween (PBS/T) and blocked with 300 μL with PierceTM protein-free (PBS) blocking buffer (Thermo Fisher Scientific, Waltham, MA, USA) for 2 h at RT. Mice sera were obtained 4 weeks or 12 weeks after single immunisation with MVA-ZIKV vaccine. Mice sera reactive to ZIKV E was serially diluted three-fold down in PBS/T with 50 μL per well as final volume and incubated for 2 h at RT. Following washing six times with PBS/T, bound antibodies were detected following a 1 h incubation with 50 μL of alkaline phosphatase-conjugated antibodies specific for whole mouse IgG (A3562-5ML, Sigma Aldrich, St. Louis, MO, USA). Following a further six washes with PBST, development was achieved using 100 μL of 4-nitrophenylphosphate diluted in diethanolamine buffer and the absorbance values at OD405 were measured and analysed using a CLARIOstar instrument (BMG Labtech, Aylesbury, GB). Serum antibody endpoint titres were defined by the sera dilution reaching a greater value than the average OD405 value of the control naïve sera plus three standard deviations.

ELISPOT was carried out using peripheral blood mononuclear cell (PBMCs) isolated from the blood as previously described [17]. Briefly, MAIP ELISPOT plates (Millipore) were coated an Anti-mouse IFN-γ mAb, contained in the Mouse IFN-γ ELISpot BASIC (ALP) kit Ref 3321-2A), after 1 h blocking with complete DMEM media (10% FCS). Isolated PBMCs (using ACK buffer solution) were plated alongside 20-mer specific peptides overlapped by 10 a.a. (10 μg/mL) and 2.5 × 10^5^ splenocytes per well. After 16 h incubation, cells were discarded and plates washed with PBS. Following this, 50 μL of biotinylated anti-mouse IFNγ mAb (1:1000 in PBS) was added to each well and incubated for 2 h. After washing, plates were incubated with 50 μL of ALP (1:1000 in PBS) reagent for 1 h. After another washing step, developing solution (Bio-Rad, Watford, UK) was used. Once spots were visible, the reaction was stopped by washing plate off with water. Spots were acquired using an ELISPOT reader. Spot forming cells (SFC)/10^6^ PBMCs producing IFNγ were calculated.

The ZIKV challenge was performed as described [26]. Briefly, naïve and vaccinated BALB/c mice (*n* = 5 per group) were infected at week four by the intravenous (i.v.) route with 10^5^ viral particles (10^2^ plaque-forming units (PFU)) of the ZIKV-BR strain. Viral loads following ZIKV challenge were quantitated by RT-PCR at day 1, 2, 3, 4, and 7. Sample size was determined to achieve 80% power to detect significant differences in protective efficacy. Vaccines were administered in a blind experiment. After the experimental outcome of the ZIKV challenge, samples were decoded.

Viral loads were assessed as described [26]. Briefly, RNA was extracted from serum with a QIAcube HT (Qiagen, Germantown, U.S.A). RNA was purified using the RNA clean and concentrator kit (Zymo Research, Irvine, U.S.A), and RNA quality and concentration was assessed by the BIDMC Molecular Core Facility of Harvard University. Log dilutions of the RNA standard were reverse-transcribed and included with each RT-PCR assay. Viral loads were calculated as virus particles per mL. Assay sensitivity was 100 copies per mL. The infectivity of virus in peripheral blood from ZIKV challenged mice was confirmed by PFU assays. Specific primers were used to amplify a region contained in the Capsid of the ZIKV genome.
